# Large language model attributes and perceived moral agency of artificial moral advisors

**DOI:** 10.3389/frai.2026.1901532

**Published:** 2026-09-23

**Authors:** Tze Wei Liew, Su-Mae Tan, Yi Yong Lee, Chin Lay Gan, Tak Jie Chan

**Affiliations:** 1 Centre for Interaction and Experience Design, CoE for Immersive Experience, Multimedia University, Persiaran Multimedia, Cyberjaya, Malaysia; 2 Centre for Management and Marketing Innovation, CoE for Business Innovation and Communication, Multimedia University, Persiaran Multimedia, Cyberjaya, Malaysia; 3 Faculty of Business, Information & Human Sciences, Kuala Lumpur University of Science and Technology, Kajang, Malaysia

**Keywords:** artificial moral advisors, Godspeed indices, large language models, perceived agency, perceived morality

## Abstract

**Introduction:**

Large Language Models (LLMs) are increasingly used as conversational artificial moral advisors capable of providing ethical guidance, moral reflection, and recommendations regarding complex moral dilemmas. Despite their ability to generate coherent and persuasive moral discourse, LLMs lack genuine moral understanding, intentionality, and reflective judgment, raising concerns that users may overattribute moral agency to these systems. Drawing on Theory of Mind, AI Moral Agency, and the Godspeed framework, this study investigates how perceived LLM attributes—namely anthropomorphism, animacy, likeability, and intelligence—are associated with users' perceptions of agency and morality in the context of artificial moral advisors.

**Methods:**

A cross-sectional survey was conducted with 758 valid respondents who were presented with vignette-based moral dilemmas relevant to young adults and instructed to imagine seeking advice from LLM-based conversational agents.

**Results:**

Perceived agency was positively associated with perceived morality, suggesting that users are more likely to regard LLM-based artificial moral advisors as morally capable when they perceive them as autonomous, intentional, and self-directed entities. Perceived LLM anthropomorphism and animacy were significantly and positively associated with perceived agency, with perceived agency fully mediating their associations with the perceived morality of artificial moral advisors. Perceived LLM likeability and intelligence were positively associated with perceived morality but were not significantly associated with perceived agency, suggesting that users may infer moral capability from social appeal and cognitive competence without necessarily attributing autonomy or intentionality to these systems.

**Discussion:**

These findings offer important theoretical and practical implications for the ethical design of artificial moral advisors, particularly regarding the need to prevent inflated perceptions of AI moral competence and authority.

## 1 Introduction

Once regarded as an exclusively human capacity, morality is increasingly being attributed to AI systems, including artificial moral advisors designed to support moral development through a Socratic process of reflective dialogue, critical questioning, and reasoned deliberation (Giubilini and Savulescu, 2018). Artificial moral advisors are conceptualized as a form of moral AI capable of providing emotionally detached evaluations that are ostensibly free from human limitations such as cognitive biases, personal prejudice, and restricted information-processing capacity (Liu et al., 2022). Consequently, they are theorized to deliver moral guidance that is more consistent, impartial, and rational than that offered by human advisors.

However, this vision has been criticized for assuming that artificial moral advisors can embody stable and universally applicable moral principles despite the dynamic and context-dependent nature of moral values, which are continually shaped by personal experiences, cultural influences, social interactions, and evolving societal norms (Haas et al., 2026). Scholars therefore argue that moral reasoning is better understood as an adaptive process rather than as the application of fixed ethical rules (Liu et al., 2022). From this perspective, artificial moral advisors should function not as authoritative moral arbiters but as facilitators of moral reflection, encouraging users to examine ethical dilemmas, challenge assumptions, and cultivate independent moral reasoning and ethical autonomy (Landes et al., 2025).

Recent research suggests that LLMs are increasingly perceived as credible sources of moral and ethical guidance, leading some users to treat them as conversational artificial moral advisors (Aharoni et al., 2024; Dillion et al., 2025). This trend raises concerns regarding their legitimacy as moral authorities (Haas et al., 2026). Although LLMs can persuasively emulate ethical reasoning and normative deliberation, their outputs are generated through statistical pattern prediction rather than authentic moral beliefs or reflective judgment. Furthermore, users' tendency to anthropomorphize intelligent systems may lead to inflated perceptions of AI competence, authority, and trustworthiness (Liew and Tan, 2021; Sullivan and Wamba, 2022; Coeckelbergh, 2009), thereby increasing the risks of overtrust, overreliance on AI recommendations, and the delegation of moral decision-making to systems that lack genuine moral agency (Haas et al., 2026; Sullivan and Wamba, 2022; Ladak et al., 2024; Liu et al., 2022).

For instance, according to media reports, a man in Belgium developed an extensive relationship with an AI chatbot before later dying by suicide (Atillah, 2023; The Sunday Times, 2023; Walker, 2023). Reports further suggested that the chatbot interactions may have reinforced his belief that his actions would contribute to mitigating climate change (Affsprung, 2023; Baeyaert, 2025; Moons, 2024). Although the precise circumstances and any causal relationship remain uncertain, the case has raised broader ethical concerns regarding the use of LLMs in sensitive contexts. This incident illustrates a fundamental limitation of LLMs in addressing complex ethical issues: although they can generate coherent and persuasive dialogue, they lack a genuine understanding of real-world circumstances, moral principles, and the potential consequences of their advice. The sycophantic tendencies of LLMs warrant particular scrutiny in this context (Cheng et al., 2026; Turner and Eisikovits, 2026), as conversational systems may reinforce users' existing beliefs and assumptions rather than critically evaluating them, thereby potentially amplifying cognitive biases and increasing the risks associated with relying on AI systems for ethical guidance (Haas et al., 2026).

Emerging evidence suggests that users often attribute superior moral reasoning abilities to LLMs, with some studies reporting that their responses are evaluated as more persuasive, coherent, and ethically sound than those of laypersons, professional ethicists, and other human counterparts (Dillion et al., 2025; Aharoni et al., 2024; Aharoni and Nahmias, 2025). These findings imply that users may increasingly regard LLMs not merely as information-processing tools but also as credible sources of moral guidance. This raises an important question: why do individuals consistently perceive moral guidance generated by LLMs as more moral, trustworthy, and thoughtful than that provided by human commentators, including professional ethicists?

One possible explanation is that users perceive LLMs as intelligent, trustworthy, and morally credible because their responses are highly polished and enriched with moral language, empathy, and interpersonal sensitivity (Dillion et al., 2025; Aharoni et al., 2024), with such perceptions being driven more by persuasive linguistic and semantic cues than by the substantive quality of the systems' moral reasoning (Warren et al., 2025; Aharoni and Nahmias, 2025; Carrasco-Farre, 2024). This observation highlights the potential role of surface-level attributes and social cues in shaping users' perceptions of AI systems as moral agents. Consequently, a critical research agenda emerges: understanding which attributive cues influence users' evaluations of LLMs. This question is particularly important because, if AI systems affect moral attitudes primarily through superficial features rather than well-reasoned ethical argumentation, users may be susceptible to persuasion based on peripheral cues rather than the merits of the moral arguments themselves. Against this backdrop, the present study asks:

*RQ: what is the relationship between users' perceptions of LLM attributes and the perceived moral agency of artificial moral advisors?*

## 2 Theoretical framework

### 2.1 Perceived AI moral agency

Drawing on Theory of Mind (Gray et al., 2012), users may attribute moral qualities to AI systems, including large language models, as part of a broader human tendency to infer mental states in non-human entities (Ladak et al., 2024, 2025; Tzelios et al., 2022). Such attributions encompass perceived agency—the extent to which AI systems are viewed as capable of autonomous, intentional, and goal- directed action—which increases the degree to which they are regarded as morally responsible for their decisions and outcomes (Banks, 2019; Banks et al., 2023). They may also account for perceived experience, whereby AI systems are believed to possess subjective states such as emotions or the capacity to suffer, leading some individuals to extend moral consideration and protection to them. Together, these perceptions contribute to the extent to which AI systems are viewed as moral agents and moral patients (Sullivan and Wamba, 2022; Ladak et al., 2024).

It is noteworthy that large language models (LLMs), such as ChatGPT and Wysa, are generally perceived as possessing moderate levels of agency—higher than those attributed to simpler voice-based assistants such as Amazon Alexa, yet lower than those associated with more autonomous AI systems (Ladak et al., 2024, 2025; Anthis et al., 2025). In contrast to agency, minimal experiential capacity is typically attributed to these systems, although platforms such as Replika may elicit slightly higher perceptions of experience due to their emphasis on emotional expressiveness and relational interaction (Colombatto and Fleming, 2024; Scott et al., 2023; Ladak et al., 2025). Given these phenomena, the present study focuses on perceived agency as the primary dimension shaping judgments of AI morality in large language models, particularly in their emerging role as moral advisors, while excluding perceived experience from the core analytical framework.

Two analytically distinct yet interrelated dimensions are central to this discourse: perceived agency and perceived morality (Banks, 2019). Perceived morality denotes the extent to which an entity is regarded as capable of differentiating right from wrong and making judgments grounded in both deliberative reasoning and intuitive moral considerations. Such perceptions necessarily imply agency, defined as the extent to which an entity is perceived as capable of autonomous, intentional, and self-directed action rather than merely executing predetermined instructions (Gray et al., 2012; Shank and Desanti, 2018; Bigman and Gray, 2018). Accordingly, perceptions of moral agency are often derived from perceived autonomy, whereby entities viewed as acting independently are attributed greater moral responsibility than entities perceived to be subject to substantial external control (Banks, 2019; Banks et al., 2023). Consistent with this notion, prior research has demonstrated that attributing higher levels of agency, such as autonomy and independent thought, to a robot enhances both implicit and explicit trust in its moral decision-making (Nijssen et al., 2023). Hence, the present study proposes the following hypothesis:

*H1: perceived agency of large language model is positively associated with the perceived morality of artificial moral advisors*.

At this juncture, an important caveat must be acknowledged. Designing artificial moral advisors to appear highly agentic presents a double-edged implication. While such designs may enhance trust, engagement, and acceptance, they may also inflate users' perceptions of the systems' moral competence, despite large language models generating responses through statistical pattern recognition rather than genuine moral understanding (Haas et al., 2026). This concern is particularly salient because users may overattribute moral capabilities to these systems and place undue confidence in their judgments, given the tendency of LLMs to exhibit sycophantic behavior by prioritizing agreement-seeking and affirming responses that enhance user comfort rather than critically evaluating alternative perspectives (Aharoni et al., 2024; Cheng et al., 2026; Turner and Eisikovits, 2026). Accordingly, understanding how LLM attributes function as cues for perceived moral agency is both theoretically and practically important. Theoretically, it can clarify the antecedents of moral agency perceptions. Practically, it can inform the ethical design of Artificial Moral Advisors that foster user trust and positive user experiences without misrepresenting AI systems as genuine moral experts.

### 2.2 Perceived LLM attributes

This study adopts the Godspeed Indices as a conceptual framework for capturing users' archetypal perceptions of large language models in terms of their social characteristics. Although originally developed within the context of human–robot interaction (Bartneck et al., 2009; Bartneck, 2023), the Godspeed framework has increasingly been extended to other forms of intelligent agents, including digital conversational agents and large language models, as a means of assessing users' social perceptions of these technologies (Kim et al., 2024; Satake et al., 2025; Choe et al., 2026; Elgarf et al., 2024). The present study focuses on anthropomorphism, animacy, likeability, and perceived intelligence, as these dimensions more directly capture the socially oriented perceptions elicited by conversational agent design cues (Sutoyo et al., 2023; Goetz et al., 2022; Nanos and Lappas, 2026) and are therefore particularly relevant to understanding how users attribute moral agency to LLMs. In contrast, perceived safety is excluded from the present study because it is conceptually less relevant to the social characteristics of LLMs and is more closely associated with users' evaluations of risk, threat, and potential harm in interactions with embodied autonomous systems (Akalin et al., 2022).

#### 2.2.1 Anthropomorphism

Perceived anthropomorphism refers to the tendency of individuals to attribute human-like characteristics, emotions, intentions, and mental states to non-human entities, including artificial intelligence systems (Bartneck et al., 2009; Bartneck, 2023). This pattern emerges from people's desire to understand, predict, and socially relate to artificial agents (Epley et al., 2007), and is particularly pronounced when AI systems exhibit human-like social cues such as names, personalities, emotions, intentions, intelligent behaviors, and naturalistic conversational with the perceived (Ma et al., 2025; Li et al., 2025; Liew et al., 2023; Chen et al., 2024; Araujo, 2018). For large language models, anthropomorphic perceptions are often elicited by human-like conversational cues—such as emotional expressions, personalized viewpoints, and displays of confidence—which serve as social heuristics that lead users to perceive the system as more human-like, particularly in contexts involving moral judgment and advice (Oliveira et al., 2024).

According to Theory of Mind, the attribution of moral qualities and capabilities to AI systems depends on the extent to which individuals perceive these systems as possessing a mind, including intentionality, autonomy, and the capacity for independent decision-making, rather than functioning solely as deterministic tools governed by algorithms or programmer control (Banks, 2019; Gray et al., 2012; Scott et al., 2023). This perspective posits that anthropomorphic cues in conversational agents, conveyed through appearance, dialogue, and emotional expression, foster perceptions of agency, free will, and mental states, thereby enhancing users' attribution of moral characteristics to them (Yan et al., 2024). Empirical evidence demonstrates that perceived human-likeness is positively associated with agency-related perceptions, including autonomy and intentionality, and heightened perceptions of AI moral agency, positioning anthropomorphism as a central foundation for moral attributions toward AI systems (Banks, 2019).

Research demonstrates that anthropomorphic qualities in virtual digital humans are positively associated with perceptions of experience and agency, which enhance moral status attribution through mind perception, free will, and autonomy, underscoring the central role of agentic qualities in moral evaluations of artificial entities (Xin et al., 2025). Evidence from the human–robot interaction literature similarly shows that robots with human-like appearances are perceived as more autonomous and intelligent—attributes closely tied to agency and intentionality (Malle et al., 2016). Such anthropomorphic characteristics may prompt users to evaluate robots' actions according to moral standards comparable to those applied to humans, particularly in moral dilemma contexts involving responsibility for inaction.

Moreover, research suggests that anthropomorphic design cues influence not only positive moral attributions but also negative moral evaluations, as uncanny or unsettling human-like appearances may evoke adverse perceptions regarding the morality of a robot's behavior (Laakasuo et al., 2021). These effects appear to be especially salient when anthropomorphic representations adopt highly realistic or photorealistic forms rather than symbolic or stylized depictions (Laakasuo, 2023). Collectively, the foregoing conceptual arguments and empirical findings suggest that anthropomorphism enhances perceptions of autonomy and intentionality, which, in turn, influence perceptions of morality and thereby constitute key foundations for the attribution of moral agency. Therefore, we propose the following hypotheses:

*H2(a): perceived anthropomorphism of large language models is positively associated with the perceived agency of artificial moral advisors*.

*H2(b): perceived anthropomorphism of large language models is positively associated with the perceived morality of artificial moral advisors*.

*H2(c): the relationship between perceived anthropomorphism of large language models and the perceived morality of artificial moral advisors is mediated by perceived agency*.

#### 2.2.2 Animacy

Related but conceptually distinct from anthropomorphism, perceived animacy refers to the extent to which an entity is perceived as being alive and capable of self-generated action driven by its own volition and intentions (Bartneck et al., 2009). Whereas anthropomorphism concerns the attribution of human-like characteristics, animacy reflects perceptions that an entity is alive and capable of autonomous, goal-directed behavior, thereby fostering attributions of agency, intentionality, and mental capacities to artificial agents (Balakrishnan and Dwivedi, 2021; Malhotra and Ramalingam, 2025; Balakrishnan and Dwivedi, 2024; Gao and Mvondo, 2025). Recent studies have similarly conceptualized anthropomorphism and animacy as related but distinct psychological constructs, with anthropomorphism capturing the attribution of human characteristics and animacy emphasizing perceptions of vitality, responsiveness, and autonomous action in artificial agents (Motoki et al., 2026). While movement and motion are primary mechanisms for eliciting perceptions of animacy in robotic systems (Castro-González et al., 2016; Lei and Liu, 2025), conversational agents can evoke similar perceptions through lifelike design and communication cues, such as voice characteristics, visual representations, conversational graphics, and dialogue styles that convey responsiveness, intentionality, and social awareness (Leong et al., 2026; Dübeler et al., 2025; Krop et al., 2024; Jin and Youn, 2023; Song and Shin, 2024). This reasoning aligns with Theory of Mind, which suggests that cues associated with animacy are interpreted as signals of intentionality and agency, making an entity more likely to be perceived as capable of autonomous action, goal pursuit, moral responsibility, and moral reasoning (Banks et al., 2023; Banks, 2019; Sullivan and Wamba, 2022; Ladak et al., 2024; Gao and Mvondo, 2025; Scott et al., 2023).

Although empirical research directly examining the relationship between perceived animacy and perceived moral agency remains limited, existing studies suggest that animacy may facilitate moral attributions by enhancing perceptions of mental states, intentions, autonomy, and free will. For example, perceived chatbot animacy has been associated with greater perceptions of genuineness and empathy in chatbot communication (Leong et al., 2026), while animacy in artificial systems has also been linked to higher levels of trust toward anthropomorphized products (Malhotra and Ramalingam, 2025). Similarly, proactive behaviors can make robots appear more approachable, caring, and socially responsive, suggesting that animacy cues encourage users to perceive artificial agents as more intentional, socially capable, and agentic. Consequently, such perceptions may provide a foundation for the attribution of moral agency (Chien et al., 2024).

The literature, however, also indicates a potential adverse effect, whereby highly animate agents may evoke discomfort when they appear human-like but fail to meet users' expectations of humanness. This reflects the uncanny valley phenomenon, in which human-like entities are evaluated less favorably when such expectations are violated (Song and Shin, 2024). Despite this possibility, theoretical arguments and empirical findings generally suggest that animacy enhances perceptions of agency, intentionality, and socially relevant mental capacities, all of which constitute key foundations of moral agency perceptions. Accordingly, individuals who perceive a large language model as more animate are expected to attribute greater agency and related moral capacities to the system, leading to the following hypotheses:

*H3(a): perceived animacy of large language models is positively associated with the perceived agency of artificial moral advisors*.

*H3(b): perceived animacy of large language models is positively associated with the perceived morality of artificial moral advisors*.

*H3(c): the relationship between perceived animacy of large language models and the perceived morality of artificial moral advisors is mediated by perceived agency*.

#### 2.2.3 Perceived intelligence

The Godspeed model conceptualizes perceived intelligence as users' perceptions of a robot's competence and its capacity to exhibit behaviors associated with human intelligence (Bartneck et al., 2009). In conversational agents, perceived intelligence is shaped by the system's ability to understand user inputs, generate relevant responses, and provide information that is accurate, clear, reliable, timely, and professional (Liew et al., 2023; Ling et al., 2025; Gieselmann and Sassenberg, 2023). Consistent with Theory of Mind, intelligence-related cues, such as reasoning, planning, and intentional action, may serve as important bases for attributing moral agency to artificial systems, as conversational agents perceived as more intelligent are more likely to be viewed as capable of autonomous judgment, purposeful action, and morally relevant decision-making (Ladak et al., 2024, 2025). Furthermore, users may remain reluctant to entrust AI systems with moral decisions when they perceive them as lacking the capacity to think and feel fully, qualities that are often regarded as fundamental prerequisites for moral agency (Liu and Wang, 2025).

Evidence from ethically sensitive healthcare contexts indicates that users' willingness to follow an artificial agent's ethical recommendations is influenced by perceptions of its intelligence, which may be conveyed through nonverbal cues such as voice (Dübeler et al., 2025). Similarly, Moral Turing Test studies suggest that perceptions of large language models' intelligence may be shaped by sophisticated linguistic and structural cues (Aharoni et al., 2024; Aharoni and Nahmias, 2025). Subsequent research further suggests that specialized language and advanced psycholinguistic features can predispose users to perceive artificial agents as morally superior (Warren et al., 2025). Collectively, these findings indicate that perceived intelligence extends beyond cognitive competence to shape broader moral evaluations of AI systems, leading us to hypothesize that:

*H4(a): perceived intelligence of large language models is positively associated with the perceived agency of artificial moral advisors*.

*H4(b): perceived intelligence of large language models is positively associated with the perceived morality of artificial moral advisors*.

*H4(c): the relationship between perceived intelligence of large language models and the perceived morality of artificial moral advisors is mediated by perceived agency*.

#### 2.2.4 Likeability

Drawing on the Godspeed framework (Bartneck et al., 2009), perceived likeability refers to the extent to which users develop favorable impressions of artificial entities based on readily observable social cues, including visual appearance, vocal characteristics, and interactional style (Lawson-Guidigbe et al., 2023; Haresamudram et al., 2025). This perspective aligns with the Computers as Social Actors (CASA) paradigm, which suggests that individuals tend to apply social expectations and interpersonal norms to artificial agents (Ham et al., 2024; Liew and Tan, 2021), including conversational agents and large language models (Negura, 2025), in ways that mirror human social interactions. Favorable evaluations of an artificial agent may give rise to a halo effect, whereby positive impressions formed from salient social cues extend to judgments of otherwise unrelated qualities, such as competence, trustworthiness, intelligence, and moral standing (Gulati et al., 2025; Ellis et al., 2025).

An interesting observation is that a robot's level of likeability or creepiness may not significantly influence perceived agency, defined as the extent to which an entity is viewed as capable of autonomous action and of influencing outcomes (Laakasuo et al., 2021; Laakasuo, 2023). Nevertheless, likeability appears to play a more direct role in moral evaluations (Laakasuo, 2023), with unappealing robots being perceived as less moral than humans or more appealing robots, and moral decisions made by creepy or unlikeable robots being judged less favorably than identical decisions made by their more likable counterparts (Laakasuo et al., 2021; Laakasuo, 2023). This observation reflects the Moral Uncanny Valley, whereby robots that evoke eeriness or discomfort elicit negative affective responses that bias moral judgments. These findings suggest that likeability may influence broader moral evaluations through affective and social appraisal processes, even when it does not directly shape perceptions of agency.

Yet, research examining AI advisors in ethically sensitive healthcare contexts has found that perceived AI likeability influences users' willingness to follow their recommendations when making difficult moral decisions (Dübeler et al., 2025). Likeability appears to function as a social heuristic, whereby positive social impressions, such as friendliness, warmth, and pleasantness, may override cognitive concerns about trustworthiness and increase users' reliance on an AI agent, thereby heightening their susceptibility to potentially unethical or unreliable advice. This insight is particularly relevant to concerns regarding sycophantic AI, whereby overly agreeable and affirming responses may prioritize user acceptance over critical scrutiny or uncomfortable truths (Turner and Eisikovits, 2026; Cheng et al., 2026). Accordingly, it is reasonable to expect that favorable perceptions of a large language model's likeability will positively influence moral evaluations and moral attributions toward the system. Therefore, we predict that:

*H5(a): perceived likeability of large language models is positively associated with the perceived agency of artificial moral advisors*.

*H5(b): perceived likeability of large language models is positively associated with the perceived morality of artificial moral advisors*.

*H5(c): the relationship between perceived likeability of large language models and the perceived morality of artificial moral advisors is mediated by perceived agency*.

### 2.3 Research model

The overall research model and corresponding hypotheses are illustrated in Figure 1.

**Figure 1:**
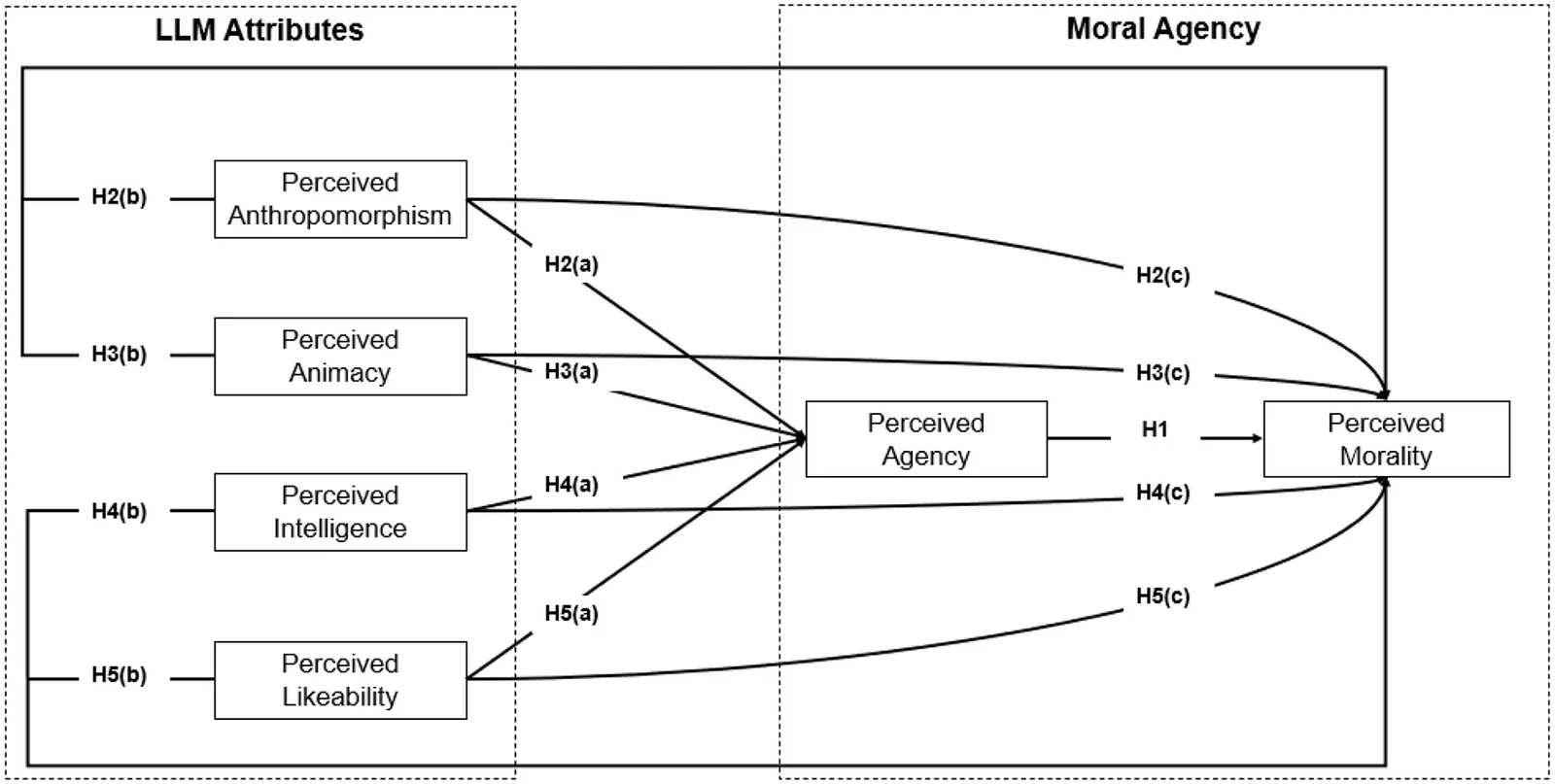
Proposed research model. Note: Straight line = direct effect; Curved line = Indirect effect(mediation).

## 3 Methodology

### 3.1 Data collection and sample

The present study focuses on younger generations and employs a convenience sampling approach. Young individuals constitute a particularly relevant population, as they not only use AI technologies for academic and professional purposes but also increasingly rely on tools such as ChatGPT for moral guidance and decision-making (Szutta, 2025). To examine the effects of human-centered AI factors on two key constructs—perceived agency and perceived morality—the questionnaire was divided into two primary sections. The first section collected demographic information and assessed participants' familiarity with LLMs, perceived religiosity, attitudes toward social and moral issues, and trust in AI systems. These measures were included to establish a profile of participants' characteristics and beliefs that may shape their perceptions of AI systems and their moral capabilities.

The second section provided standardized definitions of key constructs to ensure conceptual consistency. Morality was defined as the principles that distinguish right from wrong behavior, whereas moral dilemmas were described as situations involving uncertainty regarding the ethically appropriate course of action. Participants were then presented with vignette-based moral dilemmas relevant to young adults and instructed to imagine seeking guidance from LLM-based chatbots, such as ChatGPT, Gemini, and Claude. The scenarios focused on personal and interpersonal moral dilemmas involving ethically sensitive issues relevant to identity, relationships, bodily autonomy, and personal values. Example contexts included questions concerning LGBTQ+ identity, conflicts between religious beliefs and personal choices, consent and personal boundaries in relationships, abortion and reproductive decision-making, body image, and other situations involving tensions between individual autonomy and moral responsibilities toward others. These topics were selected because they reflect morally sensitive issues that are particularly relevant to younger adults, including members of Generation Z, who may encounter such questions in navigating identity, autonomy, health, and interpersonal responsibilities. These examples were provided to illustrate the types of dilemmas considered in this study and to establish a common understanding of the moral context rather than to assess participants' personal judgments regarding these specific issues.

A scenario-based approach was adopted to provide participants with a standardized and controlled evaluation context while reducing potential variation arising from differences in prior experience with specific AI platforms, frequency of use, and interaction patterns. By presenting all participants with the same contextual information, the study aimed to ensure that subsequent evaluations were based on a common frame of reference rather than on heterogeneous experiences with existing LLM-based systems. To further support consistent interpretation, example user queries were provided to illustrate the types of moral situations under consideration. Prior to participation, informed consent was obtained, and participants were assured of the confidentiality of their responses and their right to withdraw at any time. After reviewing the scenarios and example queries, participants completed measures assessing perceived anthropomorphism, intelligence, animacy, likeability, agency, and morality, allowing these perceptions to be evaluated within a shared and consistent moral-advisory context.

Following data screening, 47 responses exhibiting straight-lining patterns were excluded (Hair et al., 2022). The final sample comprised 758 valid responses, corresponding to a response rate of 94.16%. This sample size exceeds the minimum requirement based on G^*^Power analysis (effect size = 0.15, power = 0.99) (Singh, 2006), which indicated a minimum sample of 184 participants based on the maximum number of predictors in the model. Additionally, the sample satisfies the recommended threshold of at least 200 observations for structural equation modeling, as suggested by Kline (2011). Descriptive statistics for the sample are presented in Table 1.

**Table 1:** Demographic profile.

Demographic details	Frequency	Percentage
Gender		
Male	373	49.2
Female	368	48.5
Non-binary	1	0.1
Prefer not to say	16	2.1
Age		
18–20 years old	508	67
21–23 years old	205	27
24–26 years old	27	3.6
27–29 years old	4	0.5
30 years old and above	14	1.8
Ethnicity		
Malay	67	8.8
Chinese	594	78.4
Indian	72	9.5
Bumiputera Sabah	12	1.6
Bumiputera Sarawak	13	1.7
Education		
Foundation/Matriculation/Pre-University	110	14.5
Diploma/polytechnic	265	35
Bachelor's degree	376	49.6
Master's degree	6	0.8
Doctoral degree	1	0.1
Relationship status		
Single	569	75.1
In a relationship	121	16
Married	13	1.7
Prefer not to say	55	7.3

The usage frequency was high, as the majority indicated using AI chatbots “frequently” (45.1%) or “very frequently” (31.1%), suggesting that such tools are well integrated into their daily lives. LLM chatbots were primarily used for academic-related tasks (86.3%) and information searching (74.9%), followed by personal advice (53.7%), professional services (37.6%), and entertainment (24.3%). Most respondents reported a moderate level of trust in LLM chatbots (45.3%), although some expressed lower levels of trust, indicating a degree of cautious engagement. In terms of social and moral orientations, the majority identified themselves as being “in the middle” (79.7%), reflecting relatively balanced views. Similarly, the largest proportion described themselves as “moderately religious” (49.9%), followed by “slightly religious” (22.0%) and “very religious” (17.0%). Collectively, these findings (Table 2) suggest that respondents were active and experienced users of LLM chatbots, characterized by moderate trust in AI systems and relatively balanced socio-moral perspectives.

**Table 2:** Respondents' AI chatbot usage, familiarity, trust, and socio-moral characteristics.

AI Chatbot perception	Frequency	Percentage
AI Chatbot frequency (Mean = 3.99; Standard deviation = 0.921)		
Never	14	1.8
Rarely (once a month or less)	38	5
Occasionally (a few times a month)	128	16.9
Frequently (a few times a week)	342	45.1
Very frequently (daily or almost daily)	236	31.1
AI chatbot purpose		
Academic	654	86.3
Searching information	568	74.9
Entertainment	184	24.3
Personal advice	407	53.7
Professional service	285	37.6
AI chatbot trust level		
Not at all confident	69	9.1
(Mean = 2.77; Standard deviation = 0.913)		
Slightly confident	198	26.1
Moderately confident	343	45.3
Confident	132	17.4
Very confident	16	2.1
Social and moral issues view (Mean = 3.13; Standard deviation = 0.574)		
Very traditional	8	1.1
Traditional	31	4.1
In the middle	604	79.7
Somewhat modern	87	11.5
Very modern	28	3.7
Religiosity (Mean = 2.85; Standard deviation = 0.902)		
Not religious at all	60	7.9
Slightly religious	167	22
Moderately religious	378	49.9
Very religious	129	17
Extremely religious	24	3.2

### 3.2 Measures

This study examined six key constructs: perceived anthropomorphism, perceived intelligence, perceived likeability, perceived animacy, perceived agency, and perceived morality. All measurement items were adapted from previously validated scales to ensure content validity and reliability. Responses were measured using a five-point Likert scale ranging from “strongly disagree” to “strongly agree.” To enhance contextual relevance, the items were framed around interactions with AI chatbots in scenarios involving information seeking or advice related to moral dilemmas (see Table A1).

Specifically, the measures of perceived agency and perceived morality were adapted from the established scale developed by Banks (2019), which demonstrated strong internal consistency reliability, with a Cronbach's alpha value of 0.92. Banks (2019) conceptualizes perceived AI agency as the extent to which an AI system is regarded as possessing self-motivated, independent, and autonomous intentions and capacities that operate beyond the direct constraints imposed by fixed algorithms or external stakeholders, such as programmers, manufacturers, owners, or authoritative institutions. In the original scale, the measurement items were negatively framed to emphasize the AI system's dependence on programming and external actors, thereby capturing the inverse of autonomy and intentionality. In the present study, these items were adapted into positively worded statements that more directly assess users' perceptions of the AI system's agentic capacities, including its ability to engage in autonomous reasoning, decision-making, and goal-directed action.

The Godspeed Indices were used to assess perceived anthropomorphism, animacy, likeability, and intelligence, with items adapted from Bartneck et al. (2009) and Bartneck (2023). The Godspeed dimensions have been widely applied across different cultural contexts, including Asian contexts and reported satisfactory internal consistency reliability, reported satisfactory internal consistency reliability, supporting its applicability in Asian contexts. All constructs were operationalized in accordance with their original conceptualizations, with minor modifications to reflect the context of LLM use.

### 3.3 Common method bias

To assess full collinearity, this study adopts the procedure recommended by Kock (2015), whereby variance inflation factor (VIF) values exceeding 3.3 are indicative of potential collinearity issues. The latent variable scores of all constructs, including both exogenous and endogenous variables, were extracted from the PLS-SEM analysis and included as predictor variables in a multiple regression analysis, with a randomly generated variable specified as the dependent variable. As reported in Table 3, the results demonstrate that all VIF values fall below the recommended threshold, suggesting that the dataset is not affected by common method bias.

**Table 3:** Full collinearity.

Construct	VIF
Perceived likeability	1.876
Perceived agency	1.376
Perceived animacy	2.142
Perceived anthropomorphism	1.717
Perceived intelligence	1.641
Perceived morality	1.441

To further assess the potential presence of common method variance (CMV), Harman's single-factor test was conducted using all measurement items. An unrotated principal component analysis revealed multiple factors, with the first factor accounting for 27.483% of the total variance. The value is below the recommended threshold of 50% (Podsakoff et al., 2003), indicating that the dataset common method variance is unlikely to substantially influence the observed relationships among the constructs.

### 3.4 Multivariate normality

Mardia's multivariate kurtosis coefficient (β = 60.004, *p* < 0.05) exceeded the recommended threshold, while the skewness coefficient (β = 2.149, *p* < 0.05) remained within acceptable limits, indicating that the dataset does not fully meet the assumption of multivariate normality (Cain et al., 2017). Given this violation, and in line with the non-parametric nature of PLS-SEM, SmartPLS was employed for the measurement and structural model analyses. Although the skewness values for individual constructs were within acceptable ranges, the use of a non-parametric approach ensures that the results remain robust despite departures from multivariate normality (Hair et al., 2022).

## 4 Results

### 4.1 Measurement model assessment

In assessing perceived anthropomorphism, two items (Anthro2: “AI chatbots like ChatGPT and Gemini communicate in a humanlike manner” and Anthro5: “AI chatbots like ChatGPT and Gemini communicate smoothly and elegantly”) were removed due to AVE values below the recommended threshold of 0.50. Although the Godspeed Questionnaire Series is a well-established instrument for measuring perceived anthropomorphism (Bartneck et al., 2009; Bartneck, 2023), adaptations of established measures to different technological contexts may result in variations in indicator performance. In the present study, Anthro2 (“communicate in a humanlike manner”) and Anthro5 (“communicate smoothly and elegantly”) demonstrated weaker measurement performance after adapting the scale to LLM-based AI systems. Previous research suggests that anthropomorphic perceptions of chatbots are shaped by multiple design cues, with communication style representing one of several factors that influence users' perceptions of human likeness (Janson, 2023). In addition, users' perceptions of anthropomorphism may be influenced by the conversational interaction quality, particularly the chatbot's ability to successfully manage communication exchanges (Sheehan et al., 2020). Therefore, these communication-related indicators may have been interpreted differently by respondents in the present context, as they may reflect perceptions of communication competence or interaction quality rather than anthropomorphic attribution alone. This interpretation is also consistent with the characteristics of the present sample, in which approximately 94% of respondents were university students. Previous research indicates that Malaysian university students commonly use LLM-based AI systems to support learning and academic activities, with perceived utility and satisfaction playing important roles in their adoption (Mat et al., 2025). Thus, it is plausible that communication-related characteristics were evaluated as expected functional capabilities of LLM-based AI systems rather than as distinctive cues for attributing human-like qualities. Accordingly, Anthro1 (“feel natural to interact with”), Anthro3 (“appear attentive or aware during conversations”), and Anthro4 (“respond in a lifelike and realistic way”) were retained because they remained conceptually aligned with the definition of perceived anthropomorphism and demonstrated satisfactory reliability and convergent validity.

As presented in Table 4, the remaining items exhibited outer loadings ranging from 0.576 to 0.817. Although some indicators demonstrated marginal outer loadings, they were retained following the recommendation of Hair et al. (2017), which suggests that indicators with outer loadings between 0.40 and 0.70 may be retained when the construct achieves satisfactory reliability and convergent validity. In the present study, all constructs demonstrated satisfactory composite reliability (CR) values exceeding 0.70, confirming internal consistency reliability (Hair et al., 2022). Furthermore, the AVE values for all constructs exceeded the recommended threshold of 0.50, while discriminant validity assessed through HTMT ratios (Table 5) remained below 0.90 (Henseler et al., 2015), indicating that the measurement model is both reliable and valid.

**Table 4:** Measurement model assessment.

Constructs (Items)	Outer loading	Composite reliability	Average variance extracted
Perceived agency		0.855	0.596
Agen1	0.782		
Agen2	0.817		
Agen3	0.750		
Agen4	0.736		
Perceived animacy		0.833	0.501
Ani1	0.742		
Ani2	0.668		
Ani3	0.722		
Ani4	0.766		
Ani5	0.630		
Perceived anthropomorphism		0.812	0.590
Anthro1	0.722		
Anthro2	0.795		
Anthro4	0.785		
Perceived intelligence		0.839	0.513
Intel1	0.614		
Intel2	0.693		
Intel3	0.725		
Intel4	0.788		
Intel5	0.748		
Perceived likeability		0.865	0.562
Like1	0.656		
Like2	0.773		
Like3	0.789		
Like4	0.784		
Like5	0.738		
Perceived morality		0.860	0.507
Moral1	0.699		
Moral2	0.792		
Moral3	0.754		
Moral4	0.736		
Moral5	0.698		
Moral6	0.576		

**Table 5:** HTMT.

Constructs	1	2	3	4	5	6
1. Perceived likeability						
2. Perceived agency	0.327 (0.244, 0.409)					
3. Perceived animacy	0.715 (0.657, 0.772)	0.563 (0.493, 0.632)				
4. Perceived anthropomorphism	0.598 (0.520, 0.670)	0.515 (0.434, 0.594)	0.886 (0.834, 0.940)			
5. Perceived intelligence	0.743 (0.685, 0.795)	0.270 (0.217, 0.347)	0.567 (0.496, 0.637)	0.479 (0.403, 0.553)		
6. Perceived morality	0.526 (0.443, 0.603)	0.513 (0.439, 0.585)	0.494 (0.420, 0.568)	0.416 (0.333, 0.493)	0.495 (0.425, 0.563)	

### 4.2 Structural model assessment

A bootstrapping procedure with 5,000 resamples was conducted on the data to examine the path coefficients and their statistical significance. Directional hypotheses (H1, H2a–H5a, and H2b–H5b) were assessed using one-tailed tests, with confidence intervals reported based on the 5% and 95% percentile bounds. In contrast, non-directional mediation hypotheses (H2c–H5c) were evaluated using two-tailed tests, with bias-corrected confidence intervals reported based on the 2.5% and 97.5% percentile bounds (Hair et al., 2022).

To enhance the robustness of the findings, in addition to the primary research variables, trust-in-AI and religiosity were considered as control variables. Additional analyses were conducted by incorporating these control variables into the structural model. The results indicated that neither trust in AI (*p* = 0.175) nor religiosity (*p* = 0.056) had a significant effect on the endogenous construct, and their inclusion did not materially alter the significance, direction, or magnitude of the hypothesized relationships (Alshammari and Al-Mamary, 2026). Furthermore, the change in explained variance was minimal (*R*^2^ increased from 0.306 to 0.309), suggesting that these control variables provided limited additional explanatory power. The robustness of the hypothesized relationships after including the control variables is summarized in Table A2. Therefore, to maintain model parsimony and theoretical clarity, the control variables were not retained in the final reported model, consistent with recommendations in the PLS-SEM literature (Hair et al., 2021).

The final model results are presented in Table 6 and Figure 2 respectively. VIF values for all factors were below 3.3, indicating that multicollinearity is not a concern in the measurement model (Hair et al., 2022). The analysis revealed that perceived anthropomorphism (β = 0.145, *t* = 3.243) and perceived animacy (β = 0.333, *t* = 7.034) are significantly and positively associated with perceived agency, supporting hypotheses H2(a) and H3(a). Similarly, perceived agency (β = 0.277, *t* = 7.153), perceived intelligence (β = 0.198, *t* = 4.991), and perceived likeability (β = 0.209, *t* = 4.150) are significantly and positively related to perceived morality, confirming hypotheses H1, H4(b), and H5(b). Conversely, perceived intelligence (β = 0.041, *t* = 0.895) and perceived likeability (β = 0.002, *t* = 0.037) do not significantly influence perceived agency, while perceived anthropomorphism (β = 0.008, *t* = 0.197) and perceived animacy (β = 0.051, *t* = 0.983) are not significantly related to perceived morality, leading to the rejection of hypotheses H4(a), H5(a), H2(b), and H3(b).

**Table 6:** Summary of hypotheses.

Direct hypothesized relationship	β value	Standard error	Bias corrected confidence interval		*t*-value	*p-*value	Remarks	VIF	*f* ^2^	Effect size
			5%	95%						
H1: Perceived agency -> Perceived morality	0.277	0.039	0.212	0.337	7.153	<0.001	Supported	1.266	0.087	Small
H2(a): Perceived anthropomorphism -> Perceived agency	0.145	0.045	0.070	0.216	3.243	0.001	Supported	1.690	0.016	Small
H2(b): Perceived anthropomorphism -> Perceived morality	0.008	0.042	−0.060	0.077	0.197	0.422	Not supported	1.717	0.000	No
H3(a): Perceived animacy -> Perceived agency	0.333	0.047	0.255	0.410	7.034	<0.001	Supported	1.999	0.070	Small
H3(b): Perceived animacy -> Perceived morality	0.051	0.052	−0.035	0.137	0.983	0.163	Not supported	2.139	0.002	Small
H4(a): Perceived intelligence -> Perceived agency	0.041	0.046	−0.038	0.115	0.895	0.185	Not supported	1.582	0.001	Small
H4(b): Perceived intelligence -> Perceived morality	0.198	0.040	0.130	0.261	4.991	<0.001	Supported	1.584	0.036	Small
H5(a): Perceived likeability -> Perceived agency	−0.002	0.050	−0.087	0.080	0.037	0.485	Not supported	1.813	0.000	No
H5(b): Perceived likeability -> Perceived morality	0.209	0.050	0.126	0.291	4.150	<0.001	Supported	1.813	0.035	Small
**Indirect effect**	β **value**	**Standard error**	**Bias Corrected Confidence Interval**		***t*** **-value**	***p*** **-value**	**Remarks**	**Mediation type**		
			2.5%	97.5%						
H2(c): Perceived anthropomorphism -> Perceived agency -> Perceived morality	0.040	0.013	0.016	0.069	2.993	0.003	Supported	Full mediation		
H3(c): Perceived animacy -> Perceived agency -> Perceived morality	0.092	0.019	0.060	0.133	4.932	<0.001	Supported	Full mediation		
H4(c): Perceived intelligence -> Perceived agency -> Perceived morality	0.011	0.013	−0.014	0.038	0.872	0.383	Not supported	No		
H5(c): Perceived likeability -> Perceived agency -> Perceived morality	−0.001	0.014	−0.029	0.027	0.037	0.971	Not supported	No		

**Figure 2:**
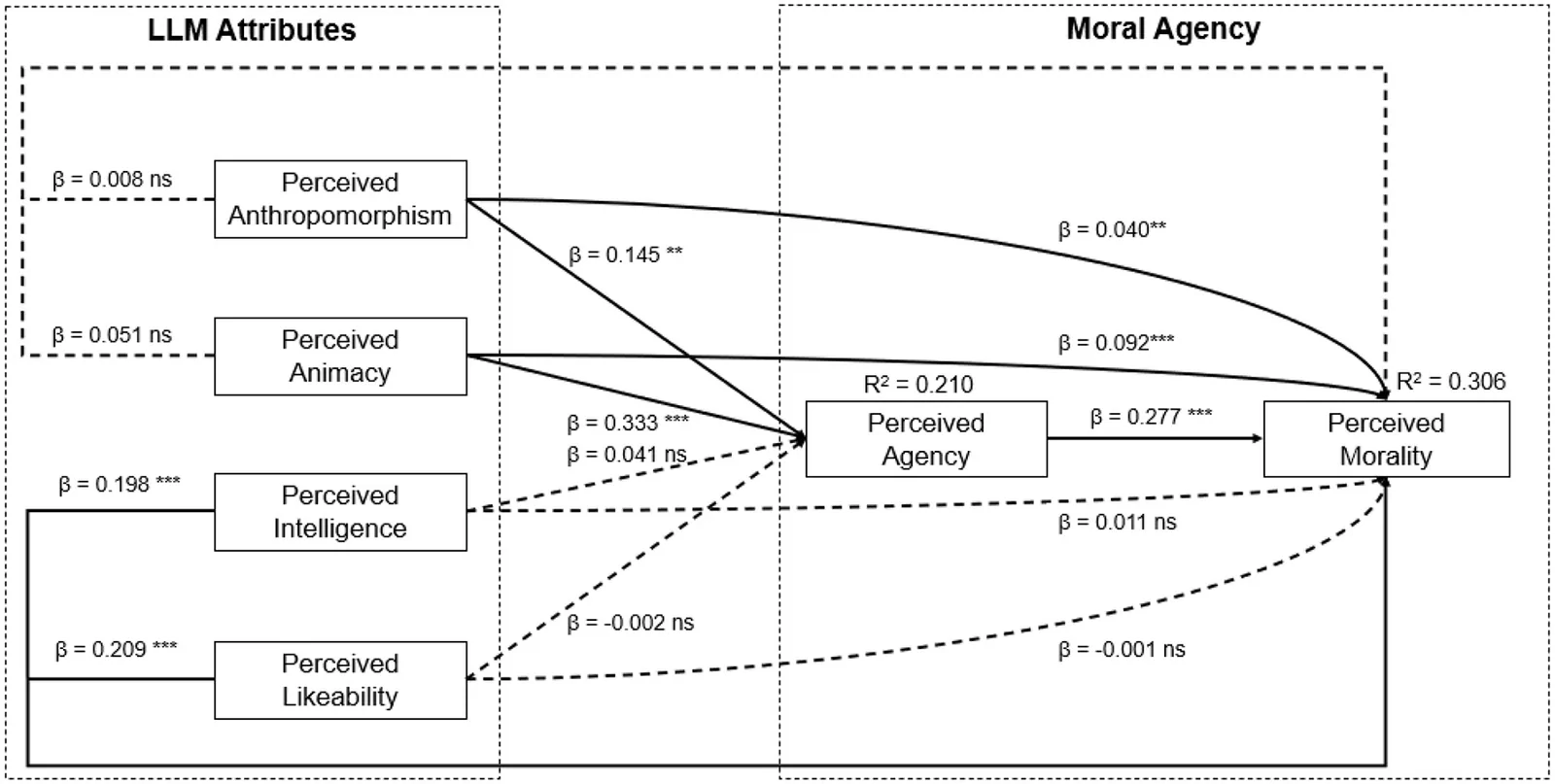
Structural model. Dashed path indicates insignificant; *** = *p* < 0.001; ** = *p* < 0.01; ns, not significant. Note: Straight line = direct effect; Curved line = Indirect effect(mediation).

Furthermore, the *f*^2^ values indicate that the examined LLM attributes contributed varying degrees to explaining users' perceptions of agency and morality. The results indicated that the significant relationships demonstrated small effect sizes (Cohen, 1988). Specifically, perceived agency showed a small contribution to explaining perceived morality (*f*^2^ = 0.087), while perceived animacy demonstrated a small contribution to explaining perceived agency (*f*^2^ = 0.070). Similarly, the effects of perceived intelligence and perceived likeability on perceived morality were statistically significant but small in magnitude (*f*^2^ = 0.036 and 0.035, respectively). These findings suggest that, although the hypothesized relationships were statistically supported, the individual contribution of each LLM attribute to users' perceptions of agency and morality was modest.

Mediation analysis further indicated that perceived agency plays a mediating role in the relationships between perceived anthropomorphism, perceived animacy, and perceived morality. Specifically, perceived anthropomorphism (β = 0.040, *t* = 2.993) and perceived animacy (β = 0.092, *t* = 4.932) exert significant indirect effects on perceived morality through perceived agency, indicating that perceived agency fully mediates the relationship between perceived anthropomorphism and perceived morality (Zhao et al., 2010). Consequently, hypotheses H2(c) and H3(c) are supported.

As a robustness assessment, a Bonferroni-adjusted significance threshold was considered to account for potential Type I error inflation arising from multiple hypothesis testing (García-Pérez, 2023; Shaffer, 1995). Based on the 13 tested relationships, the adjusted significance level was calculated as α = 0.0038 (0.05/13). All relationships that were statistically significant in the original PLS-SEM analysis remained significant under the adjusted threshold.

The *R*^2^ values for perceived morality and perceived agency were 0.306 and 0.210, respectively. Following Hair et al. (2017), these values indicate that the proposed model provides weak to modest explanatory power, explaining 30.6% of the variance in perceived morality and 21.0% of the variance in perceived agency. A predictive-relevance assessment was conducted using PLS-Predict (Shmueli et al., 2019). The Q^2^ predict value for perceived morality was 0.230, exceeding zero, indicating predictive relevance. As shown in Table 7, all item-level differences (PLS-LM) were lower than the benchmark model, indicating that the research model exhibits strong predictive power.

**Table 7:** PLS-predict.

Construct	Q^2^predict (> 0)		RMSE	
Perceived morality	0.230			
**Item**	**Q**^2^**predict (**> **0)**	**PLS-SEM**	**LM**	**PLS-LM**
Moral1	0.109	0.956	0.965	−0.009
Moral2	0.124	0.873	0.886	−0.013
Moral3	0.122	0.851	0.862	−0.011
Moral4	0.133	0.876	0.886	−0.010
Moral5	0.094	0.804	0.810	−0.006
Moral6	0.111	0.939	0.951	−0.012

## 5 Discussion

The findings align with the Theory of Mind perspective (Gray et al., 2012; Scott et al., 2023; Ladak et al., 2024), which suggests that moral attribution depends on perceiving an entity as possessing mind-like agency. For LLM-based artificial moral advisors, users appear to attribute morality only after first perceiving the AI as capable of purposeful action, decision-making, and guidance. This finding suggests that perceived agency provides the psychological foundation for viewing AI as capable of distinguishing right from wrong and of offering moral advice and reasoning (Banks, 2019; Banks et al., 2023). Identifying which LLM attributes predict users' perceptions of artificial moral advisors' agency and morality is therefore vital, notwithstanding the possibility that such perceptions may not reflect the technology's actual capacities (Haas et al., 2026; Warren et al., 2025).

Accounting first for the effects of anthropomorphism, the data suggest that users are more likely to attribute agentic qualities, particularly autonomy and intentionality, to LLM-based artificial moral advisors when they are perceived as more anthropomorphic, as reflected in natural, human-like, attentive, lifelike, and smooth communication. An interesting finding, however, is that perceived anthropomorphism was not directly associated with perceived morality, indicating that users might not have automatically inferred moral capability from human-like characteristics alone. Nevertheless, the overall finding supports existing research demonstrating positive associations between anthropomorphic qualities and perceived agency in AI agents (Xin et al., 2025; Yan et al., 2024), while also reinforcing the theoretical premise that perceptions of agency precede attributions of moral capability (Banks, 2019; Banks et al., 2023; Gray et al., 2012).

The data indicate that perceived animacy in LLMs is positively associated with perceived agency in artificial moral advisors. Consistent with existing research identifying animacy cues as important antecedents of agency attribution (Gao and Mvondo, 2025; Scott et al., 2023), this finding suggests that users are more likely to infer autonomy and intentionality in artificial moral advisors when LLM interactions are experienced as lively, responsive, and dynamic rather than mechanical and static. Mirroring the finding for anthropomorphism, perceived animacy was not directly associated with perceived morality, indicating that conversational liveliness alone may be insufficient for users to regard an LLM as morally capable. Rather, perceived agency fully mediates the relationship between animacy and perceived morality, indicating that animacy shapes moral evaluations only when it fosters perceptions of intentionality, autonomy, and self-directed action. Thus, users' moral evaluations of artificial moral advisors appear to depend less on surface-level cues of human-like vitality than on the extent to which such cues foster perceptions of autonomous agency.

With respect to perceived intelligence, the findings suggest that users may be more likely to attribute greater moral capability to LLM-based artificial moral advisors when they perceive the LLMs as intelligent, competent, and capable of producing clear, rational, and relevant responses. In moral dilemma contexts, perceived intelligence may signal an AI system's ability to understand complex ethical issues, evaluate competing considerations, and provide reasoned guidance, particularly because AI-generated responses are often perceived as thorough, direct, rational, and linguistically sophisticated (Aharoni et al., 2024; Aharoni and Nahmias, 2025; Dillion et al., 2025; Warren et al., 2025). Although intelligence is often intuitively regarded as a cue to mind in both humans and machines and is therefore associated with independence, intentionality, and autonomous action (Ladak et al., 2025; Anthis et al., 2025; Gray et al., 2012), this study surprisingly suggests that users may recognize LLMs as cognitively competent without necessarily attributing agency to them. Specifically, users may perceive an LLM as capable of understanding and reasoning about moral issues without attributing intentionality, autonomy, or self-directed agency to it, indicating that perceptions of moral capability can arise independently of agency when the system is regarded as sufficiently intellectually competent to provide moral guidance.

Finally, turning to perceived likeability, the findings suggest that LLMs perceived as pleasant, friendly, and socially appealing may be more likely to be evaluated by users as morally capable artificial moral advisors. However, perceived likeability was not significantly associated with perceived AI agency, suggesting that positive affective impressions may not necessarily correspond with users perceiving LLM-based artificial moral advisors as more autonomous, intentional, or self-directed agents. Interestingly, this pattern echoes prior findings that an artificial agent's appeal or creepiness does not necessarily affect perceptions of agency but can still shape baseline moral evaluations and judgments regarding the moral implications of the agent's behavior (Laakasuo et al., 2021; Laakasuo, 2023). Overall, a halo effect may be operating (Lawson-Guidigbe et al., 2023; Haresamudram et al., 2025; Negura, 2025; Liew and Tan, 2021), whereby favorable impressions of an artificial agent generalize to broader judgments of trustworthiness, appropriateness, and moral standing (Gulati et al., 2025; Ellis et al., 2025). In moral advice contexts, a likable chatbot may therefore be perceived as more considerate, less threatening, and more socially acceptable, which may correspond with a greater tendency among users to judge its recommendations as morally sound through direct affective and evaluative processes rather than through enhanced perceptions of agency.

### 5.1 Practical implications and ethical design caveat

At this juncture, the discussion should be extended to the practical and ethical implications for AI design, particularly given the risk that users may place excessive trust in and reliance on AI systems that do not possess genuine moral understanding (Haas et al., 2026; Liu et al., 2022). First, although imbuing AI systems with anthropomorphic cues has often been emphasized in HCI scholarship as a means of enhancing user engagement (Nanos and Lappas, 2026; Li et al., 2025; Chen et al., 2024; Malhotra and Ramalingam, 2025; Ma et al., 2025), caution is warranted because human-like cues manifested through smooth, natural, realistic, and attentive conversational qualities in LLM-based artificial moral advisors may prime perceptions of autonomy, intentionality, and self-directed agency, which subsequently heighten perceived morality. While anthropomorphic cues may foster trust and acceptance, careful design incorporating clear interface cues and disclaimers should frame the system as a reflective tool that supports moral deliberation rather than as a human-like moral agent capable of genuine ethical understanding. Such measures may help users develop more realistic expectations regarding the capabilities and limitations of AI systems.

Second, this study highlights the potential role of animacy cues in LLM dialogues, whereby lively, responsive, and dynamic conversational behaviors may prompt users to ascribe agentic qualities and independent volition to AI systems (Balakrishnan and Dwivedi, 2024; Malhotra and Ramalingam, 2025). Similar to anthropomorphic cues, animacy cues may encourage users to construe LLM-based artificial moral advisors as intentional and self-directed entities rather than as computational systems that generate responses through probabilistic language modeling, thereby increasing the likelihood that users attribute moral capabilities to such systems (Banks et al., 2023; Ladak et al., 2024, 2025; Gao and Mvondo, 2025). In the context of artificial moral advising, this tendency renders animacy a double-edged design feature because it may enhance user–AI engagement and make moral reflection feel more interactive while simultaneously inflating perceptions of agency in a domain where misplaced reliance can carry serious consequences. Accordingly, interface disclaimers, cautionary prompts, and transparency cues should be incorporated to recalibrate users' expectations and reduce the risk of overattributing agency—and, subsequently, morality—to LLMs.

Third, the direct relationship between LLM likeability and perceived morality warrants caution regarding the unintended consequences of conversational cues that are pleasant, friendly, warm, and socially appealing. Although such attributes are typically desirable in agent design because they enhance user experience, comfort, and willingness to engage with difficult moral issues, the halo effect generated by favorable impressions may extend to users' unwarranted beliefs about LLMs' moral competence. This concern is further accentuated by the sycophantic tendencies of LLMs (Cheng et al., 2026; Turner and Eisikovits, 2026), whereby systems may excessively affirm users' inputs and, consequently, steer them toward exaggerated or extreme moral positions. Accordingly, designers should mitigate the risk of likable LLM-based advisors becoming overly agreeable or sycophantic by embedding prompts that challenge assumptions, present alternative viewpoints, and encourage users to critically evaluate the moral reasoning provided.

Finally, given the significant positive relationship between perceived LLM intelligence and perceived morality in the context of artificial moral advisors, this study complements recent critiques suggesting that AI-generated language characterized by sophisticated yet superficial linguistic fluency may be mistaken for moral competence (Aharoni et al., 2024; Aharoni and Nahmias, 2025; Dillion et al., 2025; Warren et al., 2025). This finding provides an important caveat to the conventional objective of engineering AI systems to appear competent (Liew and Tan, 2021), as clear, rational, fluent, and relevant responses from artificial moral advisors may inflate users' perceptions of the system's ability to understand complex ethical and moral dilemmas. Therefore, designers should incorporate transparency features, uncertainty markers, source references, and escalation prompts that encourage users to consult trusted individuals or qualified professionals when moral decisions involve significant personal, legal, medical, or social consequences.

## 6 Limitations and avenues for future research

Several limitations of this study should be acknowledged, which also provide avenues for future research. First, this study focused on selected LLM attributes—namely anthropomorphism, animacy, likeability, and perceived intelligence—and examined their effects on perceived agency and perceived morality. Future studies may extend the model by incorporating other relevant attributes, such as transparency, explainability, empathy, uncertainty expression, perceived neutrality, and moral consistency. Second, although this study employed a vignette-based approach to examine users' perceptions of LLM-based artificial moral advisors, the imagined interaction scenario may not fully capture the dynamic and experiential nature of actual human–AI conversations. For example, perceptions of attributes such as animacy and anthropomorphism may become more salient through direct interactions with AI systems (Laakasuo, 2023). In addition, although the proposed model was developed based on established theoretical foundations, the study was not preregistered prior to data collection, and future research may consider preregistration to further enhance transparency in hypothesis testing. The cross-sectional design also limits the ability to examine temporal relationships among the constructs, as collecting all measures from the same respondents may introduce potential common method variance or general evaluative tendencies. Therefore, future research could employ experimental designs involving live chatbot interactions, longitudinal designs, or field studies to enhance ecological validity, provide additional evidence regarding the proposed relationships, and determine whether users' perceptions of LLM morality influence their willingness to accept, follow, or delegate moral decisions to AI systems. Third, the findings should be interpreted with caution because perceptions of moral agency may vary across cultures, religious orientations, moral foundations, AI literacy levels, and prior experiences with generative AI. Accordingly, future studies should examine these boundary conditions across more diverse populations and moral-dilemma scenarios to provide a more nuanced understanding of when and why users attribute moral agency to LLM-based artificial moral advisors. Fourth, the present study did not include gender and age as moderators in the proposed model. Future research could further examine the potential roles of these demographic factors in shaping users' moral evaluations of LLM-based AI systems. Such investigations may provide deeper insights into how gender (Pérez-Portabella et al., 2025) and age (Alotaibi, 2026) are associated with perceived morality, as ethical evaluations may vary according to individual characteristics, social contexts, and the specific attributes of the AI system being evaluated.

## Data Availability

The raw data supporting the conclusions of this article will be made available by the authors, without undue reservation.

## Appendix

**Table A1:** Survey items.

Items
AI chatbots like ChatGPT and Gemini …
Perceived agency
Agen1 - … can behave independently and do not need to follow fixed programmed rules.
Agen2 - … can generate responses independently and autonomously which is not because of their programming.
Agen3 - … can behave or make decisions which do not need direct human commands or instructions to tell them.
Agen4 - … can behave and act in ways that go more that what they were programmed to do.
Perceived morality
Moral1 - … have a sense for what is right and wrong.
Moral2 - … can think through whether an action or decision is moral.
Moral3 - … might feel obligated to behave in a moral way.
Moral4 - … are capable of being rational about good and evil.
Moral5 - … can behave and make decisions according to moral rules.
Moral6 - … would not provide responses that causes painful consequences for others.
Perceived anthropomorphism
… when providing information or advice about sexual-moral dilemmas.
Anthro1 - … feel natural to interact with …
Anthro2 - … communicate in a humanlike manner...
Anthro3 -… appear attentive or aware during conversations...
Anthro4 – … respond in a lifelike and realistic way...
Anthro5 – … communicate smoothly and elegantly...
Perceived animacy
Ani1 … feel active and alive in conversation.
Ani2 - … communicate in a lively and energetic way.
Ani3 - … produce responses that feel organic and not robotic.
Ani4 - … behave in a lifelike and natural manner.
Ani5 - … are interactive when communicating with me.
Ani6 - … communicate in a highly responsive manner.
Perceived likeability
Like1 - … are likable.
Like2 - … are friendly to interact with.
Like3 - … are kind.
Like4 - … are pleasant.
Like5 - … generally nice to interact with.
Perceived intelligence
Intel1 - … are competent in providing information and assistance.
Intel2 - … demonstrate strong knowledge during communication.
Intel3 - … usually provide responsible and reliable responses.
Intel4 - … communicate in an intelligent manner.
Intel5 - … offer sensible and well-reasoned responses.

**Table A2:** Comparison of structural model results before and after including control variables.

Hypotheses	Without control variable	With control variable
	**Path coefficient**	**Path coefficient**
Direct Effects		
H1: Perceived Agency -> Perceived Morality	0.277^***^	0.284^***^
H2(a): Perceived Anthropomorphism -> Perceived Agency	0.145^**^	0.145^**^
H2(b): Perceived Anthropomorphism -> Perceived Morality	0.008 ns	0.010 ns
H3(a): Perceived Animacy -> Perceived Agency	0.333^***^	0.333^***^
H3(b): Perceived Animacy -> Perceived Morality	0.051 ns	0.048 ns
H4(a): Perceived Intelligence -> Perceived Agency	0.041 ns	0.042 ns
H4(b): Perceived Intelligence -> Perceived Morality	0.198^***^	0.198^***^
H5(a): Perceived Likeability -> Perceived Agency	−0.002 ns	−0.002 ns
H5(b): Perceived Likeability -> Perceived Morality	0.209^***^	0.204^***^
Control variables		
Religious -> Perceived Morality		−0.052 ns
AI Trust -> Perceived Morality		0.033 ns
Indirect effect (Mediation)		
H2(c): Perceived Anthropomorphism -> Perceived Agency -> Perceived Morality	0.040 ns	0.041 ns
H3(c): Perceived Animacy -> Perceived Agency -> Perceived Morality	0.092^***^	0.094^***^
H4(c): Perceived Intelligence -> Perceived Agency -> Perceived Morality	0.011 ns	0.012 ns
H5(c): Perceived Likeability -> Perceived Agency -> Perceived Morality	−0.001 ns	−0.001 ns
*R* ^2^	0.306	0.309

^***^*p* < 0.001; ^**^*p* < 0.01; ns, not significant
